# The Effects of White Noise on Attentional Performance and On-Task Behaviors in Preschoolers with ADHD

**DOI:** 10.3390/ijerph192215391

**Published:** 2022-11-21

**Authors:** Hung-Yu Lin

**Affiliations:** Department of Occupational Therapy, College of Medical and Health Sciences, Asia University, Taichung 41354, Taiwan; otrlin@gmail.com

**Keywords:** ADHD, arousal, white noise, attention, hyperactivity

## Abstract

Several models have tentatively associated improving attention-deficit/hyperactivity disorder (ADHD) symptoms with arousal and external environmental stimulation. In order to further clarify the relationships between ADHD symptoms, arousal, and external stimulation, this study focused on exploring the “simultaneous” effects of white noise on intrinsic attentional performance and extrinsic on-task behaviors in preschoolers with and without ADHD. By using the computerized task (K-CPT 2), 104 preschoolers, including 52 ADHD children and 52 typically developing (TD) children, were tested and analyzed for their intrinsic attention (such as detectability, omission errors, commission errors, and reaction time). Simultaneously, these preschoolers’ external on-task behaviors were recorded for analysis through systematic observation. This study showed that white noise could effectively improve attention performance, including enhancing the ability to differentiate non-targets from targets and decreasing omission errors. It could also reduce the extrinsic hyperactive behaviors of preschoolers with ADHD. The findings of this study highlighted that white noise stimulation is a beneficial non-pharmacological treatment for preschoolers with ADHD. In contrast, for TD preschoolers, the results of this study showed that the external white noise stimuli were not only unhelpful but also a burden.

## 1. Introduction

Attention-deficit/hyperactivity disorder (ADHD) is one of the most common neurodevelopmental disorders, with a global prevalence rate of 5% in children and adolescents [1]. Of note, there is an increased prevalence among preschoolers, and several studies even found that more than 5% of children have ADHD, with an increasing prevalence among preschoolers [2]. ADHD is a behavioral disorder characterized by persistent, age-inappropriate levels of inattention, hyperactivity, and impulsivity [1]. Treatment of ADHD is an important public health issue. Multimodal treatments have been recommended for children with ADHD to decrease the risks of pharmacological treatments [3]. Based on the need for future non-pharmacological interventions to be more targeted explicitly for ADHD symptoms, non-pharmacological treatments are worth discovering and clarifying through empirical research to ensure the best intervention plan for children with ADHD.

Empirical research has investigated the arousal states of ADHD individuals over the last two decades and suggested that ADHD is related to cortical hypoarousal of the brain, which may impact cognitive performance [4]. For instance, intrinsic attentional problems in ADHD individuals have long been seen as a clinical manifestation of these arousal difficulties [5,6]. Electrophysiological studies [4,7] and fMRI meta-analyses [8,9] also support the state of hypoarousal, leading to symptoms of ADHD. Several models have assumed that patients with ADHD benefit in cognitive performance from an increase in arousal, including the cognitive-energetic model [10], the stochastic resonance (SR) effect [11], and the moderate brain arousal (MBA) model [12]. For instance, the MBA model emphasizes that the required level of additional task stimulation depends on the hypoarousal of the dopamine system so that low arousal individuals (such as children with ADHD) need more noise to achieve optimal cognitive ability than their typically developing peers. There has been a growing volume of empirical research confirming that certain types of task-irrelevant stimulation improve the cognitive performance of children with ADHD, such as background noise during memory performance tasks [12], background music during reading comprehension tasks [13], extra pictures during an auditory continuous performance test (CPT) [14], and external vestibular stimulation during visual CPT tasks [15]. Thus, external stimuli might positively affect cognitive performance, especially for individuals with ADHD.

The problem of hyperactive behaviors (such as fidgeting and restlessness) is also one of the core diagnostic features of children with ADHD [1], which can be observed very early in infancy [16]. It has also been suggested that low arousal leads to hyperactivity, which may be a self-regulating attempt to normalize tonic arousal by increasing sensory stimuli [5,17]. The optimal stimulation theory [18] states that the restless and hyperactive behavior of children with ADHD is a type of self-stimulation to increase the level of arousal and, consequently, performance. Consistent with the optimal stimulus theory, the hypoarousal model [19] assumes that children with ADHD may be drowsier than their peers and suggests that individuals with ADHD may use hyperactivity as a strategy to stay awake and be alert to counter the tendency to fall asleep. More recently, the vigilance regulation model proposed by Hegerl and Hensch [20] also claimed that children with ADHD are more prone to lower vigilance stages; the hyperactivity and sensation-seeking movements observed in these individuals may be interpreted as self-regulating attempts to create a stimulating environment for stabilizing vigilance. Although the common underlying assumption of these above models is that hyperactivity is an autoregulatory mechanism that improves the hypoarousal of individuals with ADHD; however, there is no substantial evidence to support this assumption.

Stochastic resonance (SR), a phenomenon wherein the response of a nonlinear system to a weak periodic input signal is optimized by the addition of a particular level of noise, has been widely demonstrated across various modalities [21]. The MBA model adopted this phenomenon to explain the importance of external white noise stimuli to the attentional performance of ADHD children; this model states that the SR curve is right-shifted in ADHD children due to lower dopamine and suggests that these children require more external white noise to compensate for reduced neural background activity to reach optimal brain arousal level [12]. White noise is a random mixture of audible frequencies that can improve the detection of simultaneously separated signals with equal power at each frequency. Recent research on white noise highlights many potential benefits, including improved cognition, speech comprehension, extrinsic behaviors, verbal working memory, and academic performance in individuals with ADHD [22,23,24,25,26,27]. However, not all objective methods have succeeded in showing that white noise positively affects ADHD symptoms. For example, Allen and Pammer [28] found no differences in target detection accuracy and mean reaction time under the white noise condition. Healthy individuals were also recruited by previous studies to explore the effects of white noise; however, the results were more inconsistent than studies focusing on ADHD individuals [29,30,31]. Thus, more research is warranted based on the confounding results. In addition to the inconsistent results of these white noise studies, the methodologies used to explore related issues are also inconsistent, such as adopting inconsistent auditory stimuli patterns and exploring the effects of white noise on intrinsic cognitive performance and extrinsic behavior problems separately.

Previous studies examined the effects of white noise on cognitive performance and hyperactive problems separately; these results are difficult to compare and cannot simultaneously examine the impact of white noise on the overall intrinsic and extrinsic problems in children with ADHD, especially when past studies have shown inconsistent results. Furthermore, most white noise studies recruited school-aged children or older than this age group as research subjects and rarely researched young children. To the best of my knowledge, the present study is the first to simultaneously explore the effects of white noise on intrinsic attentional performance and extrinsic on-task behaviors in preschoolers with and without ADHD. Three hypotheses were explored in this study. First, based on various symptoms exhibited by individuals with ADHD, this study hypothesized that preschoolers with ADHD would show significant differences from their TD peers in most intrinsic attentional and extrinsic on-task behavioral indicators in the condition of no background sound. Second, based on the fact that several models have tentatively associated improving ADHD symptoms with arousal and external environmental stimulation, the author hypothesized that the external sound stimulation of white noise could effectively improve the intrinsic attentional performance and extrinsic on-task behaviors of preschoolers with ADHD. Third, although the MBA model emphasizes that external sound stimulation can effectively increase functional performance for children with ADHD by improving their hypoarousal of the dopamine system, this effect may not show in TD children who already have an intact dopamine system. Therefore, the current study hypothesized that most indicators of the intrinsic attentional and extrinsic behavioral performance of TD preschoolers would not show significant differences between the conditions with and without background white noise.

## 2. Materials and Methods

### 2.1. Research Design

**Figure 1:**
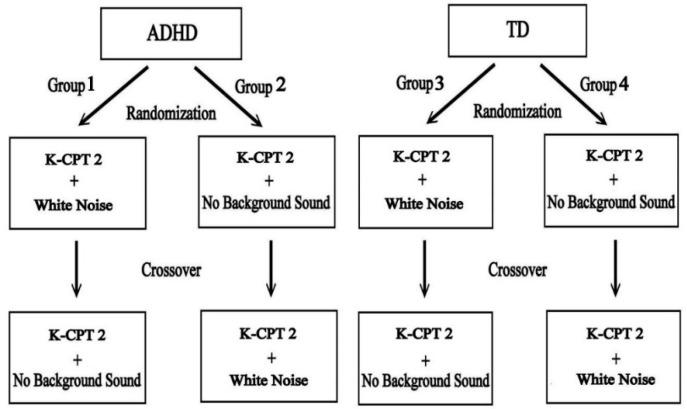
The crossover design in this study. Note: ADHD = preschoolers with attention-deficit/hyperactivity disorder; TD = typically. Developing preschoolers. K-CPT 2 = The Conners Kiddie Continuous Performance Test—Second Edition.

A two-stage randomized crossover design was applied in the present study for both children groups to balance the order of the two experimental conditions (computerized attentional test with and without background white noise) (Figure 1). The crossover design provides statistical power, as different subjects may respond to large variations in the intervention, while variation within the same subjects may be significantly less [32]. All participants had to complete the computerized attentional test twice; the crossover design could decrease the impact of learning and fatigue effects in performing this sustained attention test. Thus, the within-subjects comparison provided by the crossover design produced a more accurate estimate of the attentional performance in this study.

### 2.2. Participants

Two groups of preschoolers (children with and without ADHD) were invited to participate in this study. The group of preschoolers with ADHD was recruited first; these participants were drawn from local ADHD clinics, non-profit associations, and special education preschools. In order to form an age- and gender-matched control group, the group of preschoolers without ADHD was recruited after determining the number of eligible participants for preschoolers with ADHD; these healthy development young children were drawn from local preschools. For confirming the ability to detect differences within and between groups in this study, a minimum sample size of 84 (42 per experimental group) was calculated using the *t*-test by (G * Power, Düsseldorf, Germany), version 3.1.9 [33] with an effect size of 0.80, alpha of 0.05, and power of 0.95. A total of 104 preschoolers participated in this study: 52 participants were preschoolers with ADHD (mean age = 5.52 y), and 52 participants were typically developing preschoolers (mean age = 5.4 y) (Table 1). For the group of preschoolers with ADHD, 68 preschoolers with ADHD diagnosis signed up to participate in the early recruitment phase; however, after screening to exclude cases that did not meet the study criteria, 52 eligible preschoolers with ADHD participated in this study.

Clinical diagnoses for the group of preschoolers with ADHD were made by a licensed psychologist and two senior pediatric neurologists with more than ten years of experience in ADHD. The diagnosis procedure was conducted through a five-part process, including parent interviews, educational and medical records review, formal IQ testing (Wechsler Preschool and Primary Scale of Intelligence, WPPSI) [34], a diagnostic questionnaire inquiring about the presence of ADHD using the Diagnostic and Statistical Manual of Mental Disorders criteria (DSM-5) [1], and the Parent Rating Scales (PRS) of Behavior Assessment System for Children (BASC-3) [35] to assess symptom severity for ADHD. The Attention Problems and Hyperactivity clinical scales of BASC-3 were used to assess a subset of core ADHD symptoms. The children were confirmed as positive screens for ADHD if their T-scores were over 70 on these clinical scales. In addition, children with ADHD included in this study all met the following criteria: (1) diagnosed with ADHD by a qualified physician; (2) no psychiatric diagnoses other than ADHD; (3) full-scale IQ of WPPSI > 75; (4) without vision, hearing, and hand dysfunction (to ensure that the subjects could successfully perform the computerized attention test and make sure to receive the sound stimulus of background white noise). Participants with ADHD who are prescribed medications must undergo a minimum of 48 h of washout before testing. On the other hand, the TD children were recruited after confirming the subjects of children with ADHD participating in the present study to form the age- and gender-matched TD group. These typically developing children were recruited from community kindergartens. They all meet the following criteria: (a) no diagnosis history of ADHD and related neurological disorders; (b) attending a regular class and never attending a resource class or special education class; and (c) normal or corrected vision, hearing, and hand function. All parents or legal guardians of the participants were verbally informed of the study objectives, and their signed informed consent was collected before enrollment.

### 2.3. Setting

The experimental setting for performing computerized attentional tests was conducted in a clinic soundproofing therapy room, which contained a testing table, several chairs, computerized testing equipment, some small toys (two teddy bears and three toy cars), and a one-way mirror for test session behavioral observation. All participants had to attend the testing meeting twice in this therapy room at a one-week interval. In each appointment, every child was administered the computerized attentional test (K-CPT 2) once in this quiet room.

### 2.4. Material and Instrumentation

#### 2.4.1. White Noise

Previous studies tested several noise levels in school-age children [22,36,37]; the critical noise effect in these studies was between 70 and 80 dB. From a more theoretical perspective, to induce cross-modal stochastic resonance (SR), such as the effects of auditory stimuli on visual perception in the present study, the noise level is required to be set within the range of 73.8 ± 15.5 dB, which has an average maximum effect on the capability to recognize the sub-threshold visual stimulus [38]. Based on these empirical results, the present study’s white noise stimulus was set at 70 dB, within the “normal conversation” volume range and below what is considered harmful to hearing [39]. The white noise of rain sound, managed with an iPhone app called Muse (Webhunter Co., Ltd., Knutsford, UK, 2020), was sent to deliver binaurally via high-quality wireless kids’ headphones (Puro, San Diego, CA, USA, BT2200s) connected from an iPhone 13. In the present study, the author calibrated the decibel level of white noise using a portable digital sound level meter (Benetech, Palo Alto, CA, USA, GM1358); this white noise was not delivered for the no background sound condition.

#### 2.4.2. The Conners Kiddie Continuous Performance Test–Second Edition (K-CPT 2)

The Conners’ Continuous Performance Test (CPT) [40] and its variants are computerized attentional tests. These tests have been used for at least 60 years to measure sustained attention in many different populations. CPTs typically consist of “target” and “non-target” stimuli, presented in random order over a particular period sufficient to measure attentional performance. The Conners Kiddie Continuous Performance Test—Second Edition (K-CPT 2) [41], a type of CPT specific for assessing children ages 4 to 7, was adopted in the present study. K-CPT 2 is a task-oriented computerized assessment of attentional problems and uses pictures of objects as targets and non-targets familiar to young children. All participants were presented with a series of images on the computer screen and asked to press a button each time a target image (e.g., bicycle, train, boat) appeared and to refrain from pressing the button if the image was a soccer ball (non-target). The total test time is 7.5 min, which contains 150 targets and 50 non-targets. The K-CPT 2 reported the results on detectability, error types (omissions, commissions, perseverations), and reaction time statistics (variables related to reaction time). All computer-generated scores were reported as T-scores (mean = 50, SD = 10), with high scores reflecting severe impairment. For the aim of this study, six attentional indicators were adopted in the present study. These indicators are described below:

(1) Detectability: An indicator of inattention represents the ability to differentiate non-targets from targets;
(2) Omissions: An indicator of inattention represents the results of the failure to respond to targets;
(3) Commissions: An indicator of impulsivity represents the degree of response to non-targets;
(4) Perseverations: An indicator of impulsivity represents those that are made in less than 100 milliseconds following the presentation of a stimulus;
(5) Hit Reaction Time (HRT): An indicator of inattention or impulsivity represents the response speed of correct responses for the whole administration. An atypically slow HRT (higher T-scores) may indicate inattentiveness; alternatively, a speedy HRT (lower T-scores) may indicate impulsivity;
(6) HRT Standard Deviation (HRT SD): An indicator of inattention represents the measure of response speed consistency during the entire administration. A high HRT SD indicates a more inconsistent response speed.

#### 2.4.3. Test Session Observation: Restricted Academic Situation Scale (RASS)

Systematic direct observations of children’s behavior are essential to clinical evaluation. The Restricted Academic Situation Scale (RASS) [42], which is the most commonly used assessment tool for behavioral observation of children with ADHD during test sessions in recent decades [43], was adopted in the present study to obtain all participants’ extrinsic problematic on-task behaviors while performing the computerized attention test (K-CPT 2). RASS is a specialized coding system developed to observe and record children’s behavior when assigned tasks during a simulated independent academic situation within a clinical setting [44]. Under the coding system of RASS, the children are placed in a clinic observation room containing a table, several chairs, some toys, and a one-way mirror. Initially, the RASS was an extension of free play observations; individuals engage in an assigned academic task (a set of math problems) in playroom surroundings as a laboratory analog to classroom seatwork. In the present study, we replaced the academic task with K-CPT 2 and collected five types of problematic on-task behaviors, including (1) vocalizations, such as making meaningful or meaningless utterances; (2) off task, such as looking away from the computer monitor; (3) out of seat, such as leaving the seat; (4) fidgets, such as exhibiting repetitive motion of small movements caused by nervousness or impatience; and (5) play with objects, such as touching any object in the room unrelated to the task.

### 2.5. Procedures

Both participating groups (children with and without ADHD) were randomly assigned to two subgroups for different procedures of taking the K-CPT 2 (see Figure 1). Participants assigned to Group 1 (or Group 3) completed the K-CPT 2 under the condition of background white noise first, after which they retook the same test under the condition of no background sound one week later. Participants assigned to Group 2 (or Group 4) completed K-CPT 2 under two different background sound conditions in reverse order. The K-CPT 2 tests were administered using the standard protocol on a personal desktop computer in the clinic observation room; six attention indicators (detectability, omissions, commissions, perseverations, HRT, and HRT SD) were collected and analyzed by this computerized system. The entire 7.5 min period of on-task behaviors was recorded for each participant through the one-way mirror of the observation room, and these video files were randomly ordered before coding. The time-sampling strategy, within 15 s intervals, was applied in this study to record five types of on-task behaviors (vocalizations, off task, out of seat, fidgets, and play with objects). In every 15 s block, each task-targeting behavior was coded as one (and only one) score if the task-targeting behavior occurred during that time, regardless of how many times the behavior appeared or how long the behavior persisted in the time block.

Two trained senior therapists were blinded to research purposes, participants’ diagnoses, and group assignments and conducted behavioral coding. The two coders received detailed definitions of behavior types and were trained on practice videos. In addition, to create a blind context when coding the on-task behaviors, all subjects had to wear headphones to perform the computerized attention test regardless of the test situation (no sound stimuli were delivered through headphones under the condition of “no background sound”). Only the author, who set the K-CPT 2 and white noise settings, knew whether the white noise was delivered. This procedure created the blind condition to reduce bias on the part of the coders. The interobserver agreement (IOA), which was collected from K-CPT 2 task practice sessions in the present study, was calculated by dividing the number of intervals in agreement by the sum of agreements and disagreements and then multiplying by 100 [45]. According to the standard set by Bakeman and Gottman [46], the IOA values (k coefficient) of this study were very good (vocalizations = 0.96; off task = 0.92; out of seat = 0.98; fidgets = 0.94; play with objects = 0.98).

### 2.6. Statistical Analyses

The data of intrinsic attentional performance (K-CPT 2 testing scores) and extrinsic on-task behaviors (the data collected from test session observations) were analyzed with the statistical software package SPSS Version 21 (IBM Corp., Armonk, NY, USA). All data analyses were two-tailed, and the significance was set at *p* < 0.05. An independent sample *t*-test was used to compare the data between the two children groups, and a paired sample *t*-test was adopted to compare the data between the two background sound conditions for each children group. Cohen’s *d* was adopted to calculate the effect size, a standardized quantitative index representing the magnitude of change that one variable produces in another variable as reflected in the difference between two means independent of sample size [47]. The interpretation of effect size *d* is based on the convention proposed by Cohen, such as 0.20 being the small effect size, 0.50 being the medium, and 0.80 or more being the large effect size. The statistical significance in this study was sufficient to explain most cases only if the effect size *d* was considered a large magnitude.

## 3. Results

### 3.1. Descriptive Statistics

The main demographic information for the sample is reported in Table 1. Comparisons made using *t*-tests showed that there was no significant difference between groups in terms of age (*t* = 1.46, *p* = 0.15) and IQ (*t* = −1.55, *p* = 0.13). In addition, the typically developing children (*n* = 52) were selected to match the children with ADHD (*n* = 52) for age and gender. Both preschooler groups in the present study were well-matched concerning age, IQ, gender, and grade of schooling in preschool.

### 3.2. ADHD and TD Children’s Performance in “No Background Sound” Condition

As illustrated in Table 2, most indicators of intrinsic attentional performance, including detectability (*t* = 2.89, *p* < 0.05), omissions (*t* = 3.37, *p* < 0.05), commissions (*t* = 3.29, *p* < 0.05), perseverations (*t* = 3.11, *p* < 0.05), and HRT SD (*t* = 4.61, *p* < 0.001), showed a significant difference between groups. Similarly, most extrinsic behavioral indicators of ADHD participants, including vocalizations (*t* = 4.65, *p* < 0.001), off task (*t* = 4.75, *p* < 0.001), out of seat (*t* = 2.09, *p* < 0.05), and fidgets (*t* = 8.63, *p* < 0.001), also showed significantly worse performance than their TD peers.

### 3.3. ADHD and TD Children’s Performance in “White Noise” Condition

In the white noise condition (see Table 2), most indicators (10 out of 11) of intrinsic attentional performance and extrinsic behavioral indicators showed no significant difference between groups. Only the indicator of fidgets (*t* = 3.24, *p* < 0.05) showed a significant difference between groups; however, this difference was not sufficient to explain most preschoolers in this study (*d* < 0.8).

### 3.4. ADHD Children’s Performance in “White Noise” Condition and TD Children’s Performance in “No Background Sound” Condition

As illustrated in Table 3, most indicators (9 out of 11) of intrinsic attention and extrinsic behaviors showed no significant difference between groups. Only two indicators, omissions (*t* = 3.18, *p* < 0.05) and fidgets (*t* = 4.94, *p* < 0.001), showed significant differences between groups.

### 3.5. Comparison of ADHD Preschoolers’ Performance between Two Background Sound Conditions

The results from the paired *t*-testing revealed that half indicators, including detectability (*t* = −2.03, *p* < 0.05), omissions (*t* = −2.97, *p* < 0.05), and HRT SD (*t* = −3.64, *p* < 0.05), of intrinsic attentional performance in ADHD preschoolers between two auditory conditions were statistically significant (see Table 4). Additionally, most indicators of extrinsic on-task behavior, including vocalizations (*t* = −3.35, *p* < 0.05), off task (*t* = −4.6, *p* < 0.001), out of seat (*t* = −2.03, *p* < 0.05), and fidgets (*t* = −5.2, *p* < 0.001) of these ADHD preschoolers, showed significant differences between two background sound conditions.

### 3.6. Comparison of TD Preschoolers’ Performance between Two Background Sound Conditions

For typically developing preschoolers, white noise presented during taking computerized attentional tasks had no effect on intrinsic attentional performance (see Table 5). All six intrinsic attentional indicators, including detectability (*t* = 1.26, *p* > 0.05), omissions (*t* = 1.04, *p* > 0.05), commissions (*t* = 1.88, *p* > 0.05), perseverations (*t* = 1.53, *p* > 0.05), HRT (*t* = 1.16, *p* > 0.05), and HRT SD (*t* = 1.85, *p* > 0.05) showed no significant difference between two sound conditions. Similar results were also presented on extrinsic on-task behavior; all five extrinsic behavioral indicators, including vocalizations (*t* = 1.94, *p* > 0.05), off task (*t* = 1.21, *p* > 0.05), out of seat (*t* = 0.73, *p* > 0.05), fidgets (*t* = 1.44, *p* > 0.05), and play with objects (*t* = 0.70, *p* > 0.05), showed no significant difference between two background sound conditions.

## 4. Discussion

Several methodological advantages over previous white noise research were adopted in the present study. First, a two-period crossover design was used in the present study for two groups of children to balance the order of testing under two sound conditions; this methodologic design decreased the impact of fatigue effects in performing two sufficiently long laboratory tests, especially for the little kids. Second, because age and gender truly affect attentional performance [48,49], the author recruited age- and gender-matched TD peers in the present study to decrease the influence of these attributes. Third, the replicated, standardized, and computerized attention test, K-CPT 2, was adopted in this study to measure all participants’ intrinsic attentional performance, which permitted us to obtain objective outcomes that were not susceptible to the measurement. Last, a blind design was adopted in the coding process for all collected on-task behavioral data to minimize possible bias. Based on these strengths over previous research on related topics, the findings of this study should be valuable in discussing the effects of white noise on preschoolers with and without ADHD.

### 4.1. The Intrinsic and Extrinsic Symptoms of Preschoolers with ADHD

In the present study, we considered the attentional function and on-task behaviors of TD preschoolers in the no background sound condition as a benchmark for appropriate performance in this age group. Based on comparisons with TD preschoolers, the research results (see Table 2) support the first hypothesis in the present study in that most intrinsic and extrinsic dimensions, including five attentional indexes (detectability, omissions, commissions, perseverations, and HRT SD) and four behavioral indicators (vocalizations, off task, out of seat, and fidgets), showed significant differences between the two children groups in no background sound condition.

Further analysis of the effect size obtained in this study was conducted (see Table 2). In the condition of no background sound, there is a difference at baseline between the groups, with large effect sizes, including vocalizations (*d* = 0.91), off task (*d* = 0.93), and fidgets (*d* = 1.69). However, only the index of HRT SD (*d* = 0.90) obtained large effect sizes among attentional indexes. Furthermore, although most attention indexes showed significance between the two groups in the condition of no background sound, five of six attentional indexes showed that the ADHD sample was not 1 SD above the mean. These results were mainly influenced by the presentations of participants with ADHD in this study. Most of the preschoolers with ADHD recruited in this study were categorized into the hyperactive–impulsive presentation (51.9%) based on DSM-5 (see Table 1); this represented more than half of the ADHD participants with significant hyperactivity problems but only mild attention problems. This analysis confirmed two essential traits of ADHD preschoolers’ symptoms: (1) For preschoolers with ADHD, the problems of extrinsic hyperactive behavior are more representative than intrinsic attentional difficulties of this age bound. Compared to the hyperactive and impulsive symptoms of ADHD at this early stage of development, the signs of inattention appear late in childhood [50]. Indeed, according to previous empirical studies, the predominantly hyperactive–impulsive presentation is more common in preschool kids than in older children [51,52]. These developmental trends of ADHD symptoms are also reflected in prevalence studies using the DSM diagnostic criteria, which suggest that hyperactive–impulsive and combined type ADHD are the two most common presentations of preschoolers with ADHD, with fewer children meeting the purely inattentive DSM criteria [53]. (2) In intrinsic attentional performance, further analysis highlights the importance of performance variability (HRT SD) in preschoolers with ADHD when taking attention tasks. For minors with ADHD, previous studies suggest that school-aged children and adolescents with ADHD are consistently inconsistent on a variety of tasks, including tasks measuring reaction time on attention, motor control, choice decision, behavioral inhibition, and cognitive interference [54,55,56]. The findings of this study extended the age frame of this consistently inconsistent phenomenon to younger preschool kids.

### 4.2. The Effects of White Noise on Preschoolers with ADHD

The performance of ADHD preschoolers in the “white noise condition” and the performance of TD preschoolers in the “no background sound condition” were compared in this study (see Table 3). Based on Table 2, in the condition of no background sound (the comparison at baseline), there were 9 out of 11 indicators that were different between the groups; however, according to Table 3, there were only 2 out of 11 indicators that were still differences between the groups. These results showed that white noise brought seven of the attentional and behavioral indicators into the range of TD children. These results supported that the external environmental stimulation of white noise is a useful non-pharmacological method to improve intrinsic attentional performance and extrinsic on-task behaviors in preschoolers with ADHD. Furthermore, the performance of preschoolers with ADHD in two background sound conditions was also compared in this study (see Table 4). According to Table 4, more evidence supports white noise as an effective intervention for preschoolers with ADHD. In terms of intrinsic attentional performance, the intervention of white noise significantly reduces the tendency to ignore visual stimuli (omission) and effectively improves the variability of reaction time (HRT SD). These improvements further show that the intervention of white noise can effectively enhance the alertness of preschoolers with ADHD. On the other hand, the results of this study also show that providing white noise stimuli can effectively decrease the hyperactive behaviors in ADHD preschoolers. It is worth mentioning that two external behaviors, “Off task” and “Fidgets”, are not only showing significant differences but can also be interpreted as positive changes in most preschoolers with ADHD (*d* > 0.8, see Table 4); such large effect sizes were not shown in effect on intrinsic attentional performance. These results show that white noise has a more significant and broader positive impact on extrinsic behavioral problems in preschoolers with ADHD than on their intrinsic attentional difficulties. However, readers should apply these results with caution based on the presence of significant hyperactive and impulsive problems in this study’s sample, which may contribute to the significant impact of white noise on the hyperactive/impulsive symptoms of ADHD in this sample of preschoolers.

Theoretically, this research supports the MBA model, especially when the results showed improvements in attentional performance and hyperactive behaviors from white noise. The MBA model adopted stochastic resonance (SR), a phenomenon in which a signal that is usually too weak to be detected by a sensor can be boosted by adding white noise to the signal, to explain how white noise improves the performance of children with ADHD [11,12,21]. This study’s results indirectly supported that white noise sound stimulation can enhance visual and motor control signals across modalities in children with ADHD to improve their visual attentional performance and hyperactive behaviors.

### 4.3. The Effects of White Noise on TD Preschoolers

Regarding TD preschoolers (see Table 5), although the results of this study showed no significant difference between the two background sound conditions, all indicators exhibited a trend toward worsening attentional performance and on-task behaviors after providing white noise stimulation. Under the white noise condition, the comprehensive negative effects and marked agitation of TD preschoolers show that this external sound stimulus is not only unhelpful but also a burden, especially when they are working on attention tasks. This result also corroborates studies on children of other age groups. For instance, studies focusing on school-aged children with and without ADHD [57,58] investigated the relationship between fidgeting and cognition by asking them to perform cognitive tasks while monitoring their levels of physical activity; both studies found a positive correlation between activity level and task performance in children with ADHD but not in the TD group. The negative impact of external environmental stimulation in TD children may be explained by citing the Yerkes-Dodson law [59], which describes an inverted-U relationship between arousal and behavioral performance. Briefly, the Yerkes-Dodson law states that a high level of arousal can improve performance with easy tasks or worsen it with challenging tasks. Taking a computerized attention test, in which subjects should focus on achieving a specific goal, is seen as a challenging task for TD preschoolers with an adequate level of arousal. According to the Yerkes-Dodson law, they should avoid excessive stimulation, such as providing white noise, in challenging attention tests.

### 4.4. Limitations and Future Research

The main strength of this study lies in simultaneously exploring the effects of white noise on intrinsic attentional performance and extrinsic hyperactive behaviors for preschoolers with ADHD, which has not been examined in past research. However, there are also several limitations. First, the author only recruited ADHD preschoolers from clinical settings, and this clinic sampling limits the generalizability of this study’s results. Thus, future research is needed to collect more representative samples by recruiting community participants. Second, this study sample only included preschool children (4–6 years old), so results should not be extrapolated to children outside this age group. Third, only one type of external sound stimulation was used in the present study. Certain types of music may also positively affect ADHD patients. For example, recent studies [13,60] confirmed that listening to calm or classical music could decrease negative mood and improve reading comprehension in the ADHD group. In the future, different types of sound stimulation (e.g., classical music) should be compared with white noise to find the most appropriate and harmless non-pharmacological treatment for individuals with ADHD. Lastly, this study only investigated immediate performance on attention and on-task behaviors after receiving white noise stimuli. It would also be interesting to examine further the long-term effects of white noise on academic performance in the future.

## 5. Conclusions

In conclusion, the findings of this study highlight that white noise stimulation is a beneficial non-pharmacological treatment for preschoolers with ADHD. Furthermore, this study’s results confirmed that white noise improves not only intrinsic attentional performance (such as decreasing omission errors and reaction time variability) but also decreases extrinsic hyperactive behaviors (such as severe fidgeting). However, these significant benefits of white noise in preschoolers with ADHD were not present in their TD peers. For TD preschoolers, although the results of this study showed no significant difference between the two background sound conditions, the stimulation of white noise presented a trend toward worsening attentional performance and on-task behaviors in these TD preschoolers.

## Figures and Tables

**Table 1:** Demographic characteristics of participants.

Attribute Category	ADHD (N = 52)	TD (N = 52)	*t*	*p*
Mean age (SD)	5.52 (0.43)	5.40 (0.39)	1.46	0.15
Mean IQ (SD)	105.94 (9.26)	108.35 (6.33)	−1.55	0.13
Gender, N (%)				
Male	35 (67.3%)	35 (67.3%)		
Female	17 (32.7%)	17 (32.7%)		
Education, N (%)				
Pre-Kindergarten	11 (21.2%)	11 (21.2%)		
Kindergarten	41 (78.8%)	41 (78.8%)		
ADHD presentation, N (%)				
Inattentive Presentation	4 (7.7%)			
Hyperactive–Impulsive Presentation	27 (51.9%)			
Combined presentation	21 (40.4%)			

**Table 2:** Comparison of intrinsic and extrinsic performance between ADHD and TD preschoolers.

	White Noise ADHD (*n* = 52)/TD (*n* = 52)	NBS ADHD (*n* = 52)/TD (*n* = 52)	*t* (WN/NBS)	Cohen’s *d* Effect Size (WN/NBS)
Attention, M (SD)				
Detectability	54.12 (5.66)/54.23 (6.38)	56.37 (6.83)/52.71 (6.04)	−0.10/2.89 *	0.02/0.57
Omissions	54.02 (4.85)/54.29 (7.70)	56.94 (6.14)/53.21 (5.10)	−0.21/3.37 *	0.04/0.66
Commissions	57.27 (6.24)/55.62 (7.03)	57.63 (6.96)/53.52 (5.76)	1.27/3.29 *	0.25/0.64
Perseverations	55.56 (6.21)/54.90 (8.19)	56.33 (5.42)/53.12 (5.09)	0.46/3.11 *	0.09/0.61
Hit Reaction Time	55.42 (6.33)/56.98 (7.58)	56.73 (8.71)/55.63 (4.93)	−1.14/0.79	0.22/0.16
HRT SD	53.71 (5.41)/53.85 (6.65)	57.79 (8.22)/51.85 (4.35)	−0.11/4.61 **	0.02/0.90
On-Task Behavior, M (SD)				
Vocalizations	2.31 (1.21)/2.38 (1.26)	3.13 (1.50)/1.92 (1.14)	−0.32/4.65 **	0.06/0.91
Off task	4.31 (1.28)/4.56 (1.65)	5.69 (1.98)/4.25 (0.95)	−0.86/4.75 **	0.17/0.93
Out of seat	0.62 (0.77)/0.62 (0.72)	0.83 (0.81)/0.54 (0.58)	0.01/2.09 *	0.01/0.41
Fidgets	7.63 (1.88)/6.48 (1.75)	9.87 (2.87)/5.98 (1.52)	3.24 */8.63 **	0.63/1.69
Play with objects	0.63 (0.77)/0.75 (1.53)	0.90 (1.19)/0.60 (0.69)	−0.49/1.61	0.10/0.31

Note: WN = White noise condition; NBS = No background sound condition. Intrinsic attentional variables are presented as T-scores (higher scores reflecting more impairment). Extrinsic behavioral variables are presented as occurrence times. HRT SD = HRT Standard Deviation. * *p* < 0.05, ** *p* < 0.001.

**Table 3:** Comparison of intrinsic and extrinsic performance between ADHD preschoolers in “white noise” condition and TD preschoolers in “no background sound” condition.

	ADHD (*n* = 52) (White Noise)	TD (*n* = 52) (No Background Sound)	*t*	Cohen’s *d* Effect Size
Attention, M (SD)				
Detectability	54.12 (5.66)	52.71 (6.04)	1.22	0.24
Omissions	54.02 (4.85)	53.21 (5.10)	0.83	0.16
Commissions	57.27 (6.24)	53.52 (5.76)	3.18 *	0.62
Perseverations	55.56 (6.21)	53.12 (5.09)	1.97	0.43
Hit Reaction Time	55.42 (6.33)	55.63 (4.93)	−0.19	0.04
HRT SD	53.71 (5.41)	51.85 (4.35)	1.94	0.38
On-Task Behavior, M (SD)				
Vocalizations	2.31 (1.21)	1.92 (1.14)	1.67	0.33
Off task	4.31 (1.28)	4.25 (0.95)	0.26	0.05
Out of seat	0.62 (0.77)	0.54 (0.58)	0.58	0.12
Fidgets	7.63 (1.88)	5.98 (1.52)	4.94 **	1.02
Play with objects	0.63 (0.77)	0.60 (0.69)	0.27	0.04

Note: Intrinsic attentional variables are presented as T-scores (higher scores reflecting more impairment). Extrinsic behavioral variables are presented as occurrence times. HRT SD = HRT standard deviation. * *p* < 0.05, ** *p* < 0.001.

**Table 4:** Comparison of ADHD preschoolers’ performance between two background sound conditions.

	ADHD (*n* = 52)		*t*	Cohen’s *d* Effect Size
	White Noise	No Background Sound		
Attention, M (SD)				
Detectability	54.12 (5.66)	56.37 (6.83)	−2.03 *	0.36
Omissions	54.02 (4.85)	56.94 (6.14)	−2.97 *	0.53
Commissions	57.27 (6.24)	57.63 (6.96)	−0.42	0.05
Perseverations	55.56 (6.21)	56.33 (5.42)	−0.83	0.13
Hit Reaction Time	55.42 (6.33)	56.73 (8.71)	−1.20	0.17
HRT SD	53.71 (5.41)	57.79 (8.22)	−3.64 *	0.59
On-Task Behavior, M (SD)				
Vocalizations	2.31 (1.21)	3.13 (1.50)	−3.35 *	0.60
Off task	4.31 (1.28)	5.69 (1.98)	−4.60 **	0.83
Out of seat	0.62 (0.77)	0.83 (0.81)	−2.03 *	0.27
Fidgets	7.63 (1.88)	9.87 (2.87)	−5.20 **	0.92
Play with objects	0.63 (0.77)	0.90 (1.19)	−1.79	0.27

Note: Attentional variables are presented as T-scores (higher scores reflecting more impairment). Behavioral variables are presented as occurrence times. HRT SD = the standard deviation of hit reaction time (HRT). * *p* < 0.05, ** *p* < 0.001.

**Table 5:** Comparison of TD preschoolers’ performance between two background sound conditions.

	TD (*n* = 52)		*t*	Cohen’s *d* Effect Size
	White Noise	No Background Sound		
Attention, M (SD)				
Detectability	54.23 (6.38)	52.71 (6.04)	1.26	0.24
Omissions	54.29 (7.70)	53.21 (5.10)	1.04	0.17
Commissions	55.62 (7.03)	53.52 (5.76)	1.88	0.33
Perseverations	54.90 (8.19)	53.12 (5.09)	1.53	0.26
Hit Reaction Time	56.98 (7.58)	55.63 (4.93)	1.16	0.21
HRT SD	53.85 (6.65)	51.85 (4.35)	1.85	0.36
On-Task Behavior, M (SD)				
Vocalizations	2.38 (1.26)	1.92 (1.14)	1.94	0.38
Off task	4.56 (1.65)	4.25 (0.95)	1.21	0.23
Out of seat	0.62 (0.72)	0.54 (0.58)	0.73	0.12
Fidgets	6.48 (1.75)	5.98 (1.52)	1.44	0.31
Play with objects	0.75 (1.53)	0.60 (0.69)	0.70	0.13

Note: Attentional variables are presented as T-scores (higher scores reflecting more impairment). Behavioral variables are presented as occurrence times. HRT SD = the standard deviation of hit reaction time (HRT).

## Data Availability

The study materials and the detail of all analyses are available from the corresponding author upon reasonable request.
